# COVID-19 vaccination and unemployment risk: lessons from the Italian crisis

**DOI:** 10.1038/s41598-021-97462-6

**Published:** 2021-09-17

**Authors:** Valentina Pieroni, Angelo Facchini, Massimo Riccaboni

**Affiliations:** IMT School for Advanced Studies Lucca, Lucca, Italy

**Keywords:** Statistical physics, thermodynamics and nonlinear dynamics, Statistics, Complex networks

## Abstract

This paper analyzes the impact of mobility contraction on employee furlough and excess deaths in Italy during the COVID-19 crisis. Our approach exploits rainfall patterns across Italian administrative regions as a source of exogenous variation in human mobility to pinpoint the causal effect of mobility restrictions on excess deaths and furlough workers. Results confirm that the first countrywide lockdown has effectively curtailed the COVID-19 epidemics restricting it mainly to the northern part of the country, with the drawback of a countrywide increase in unemployment risk. Our analysis points out that a mobility contraction of 1% leads to a mortality reduction of 0.6%, but it induces an increase of 10% in Wage Guarantee Funds allowed hours. We discuss return-to-work policies and prioritizing policies for administering COVID-19 vaccines in the most advanced stage of a vaccination campaign when the healthy active population is left to be vaccinated.

## Introduction

The beginning of the vaccination campaign in response to the COVID-19 pandemic is considered the first fundamental step in recovering from the damages of the spread of coronavirus (SARS-CoV-2) worldwide. The epidemic has triggered a combined health and socio-economic crisis, with severe consequences: in 2020, the world economy shrank by 4.3%^1,2^. On the supply side, social distancing measures issued by national governments placed workers under stay-at-home orders, shut down ’non-essential’ activities, and challenged supply chains. On the demand side, the pandemic has reduced consumer spending, virtually wiping out demand in entire economic sectors such as hospitality, tourism, and travel sectors.

Vaccine strategic distribution plans generally follow the WHO guidelines^3^ and, in line with the scientific literature^4–6^, EU countries set rules to prioritizing population according to multiple risk criteria related to age, work, and health vulnerability. Guidelines usually do not consider the low-risk healthy population composing a large part of the working force, putting a consistent share of the population on the same ground in the staging of the vaccination rollout.

Here we propose a criterion for administering COVID-19 vaccines in the most advanced stage of vaccination campaigns. As a driving principle, we propose the return-to-work facilitation for the beneficiaries of wage guarantee schemes and workers facing a high unemployment risk, with the expected benefit of a more efficient allocation of public funds and a reduction of future job losses. We also provide evidence on the effectiveness of mobility restrictions for the Italian case implementing an instrumental variable (IV) framework. From the public health point of view, our findings show that a one-percent drop in mobility implies a 0.6 percent drop in excess deaths in the following month. On the other hand, a 1% drop in human mobility corresponds to a 10% increase in the Wage Guarantee Fund (WGF) in the next month. We exploited weather shocks as a source of exogenous variation in human mobility patterns to provide consistent estimates and overcome endogeneity issues. This approach is consistent with the econometric strategy followed in several empirical studies^7–9^ that exploited a naturally occurring source of variation, like the one observed in weather patterns, to infer causal relationships between variables in an IV setting.

The criterion of prioritizing workers at high unemployment risk is also compared to an alternative one based on the working population. The comparison shows that prioritizing workers based on unemployment risk exposure benefits people at a higher risk of marginalization in urban areas.

From July 2021 on, a new EU Regulation (The EU Digital COVID Certificate Regulation) allows European citizens to obtain a COVID-19 Certificate, which in principle should facilitate free movements across EU member states. Following the guidelines, some European countries are introducing the COVID-19 Certificate not just for traveling purposes but also as a requirement to enter indoor public spaces, attend events or get access to restaurants. The enforcement of the new measures has started a debate on extending the COVID-19 Certificate as a requirement to come back safely to the workplace. In this vein, Italy has already made the Certificate compulsory for school and university personnel. As non vaccinated workers in more professional categories and countries could be potentially impacted by similar restrictions in the near future, the need to account for people’s employment status and unemployment risk in delivering vaccine doses gets even more relevant.

## Results

### Effects of lockdown

Right after the pandemic outbreak in Italy^10^, the Italian government has extended by decree (Decree Law n. 18/2020 issued on March 17) the extant wage guarantee schemes against the pandemic crisis to strengthen employment protection. The National Social Insurance Agency (INPS) and Bank of Italy^11^ reported that, from March to April 2020, around $50\%$ of employers in the private sector had been allowed to use wage compensation schemes according to the new rules in force.

The growth of wage supporting schemes by the employers observed from March can be partially explained in light of an additional labor market measure issued in March, the firing freeze, i.e., a temporary suspension of firings, that dropped sharply as compared to their average level in 2017–2019^12^.

**Figure 1:**
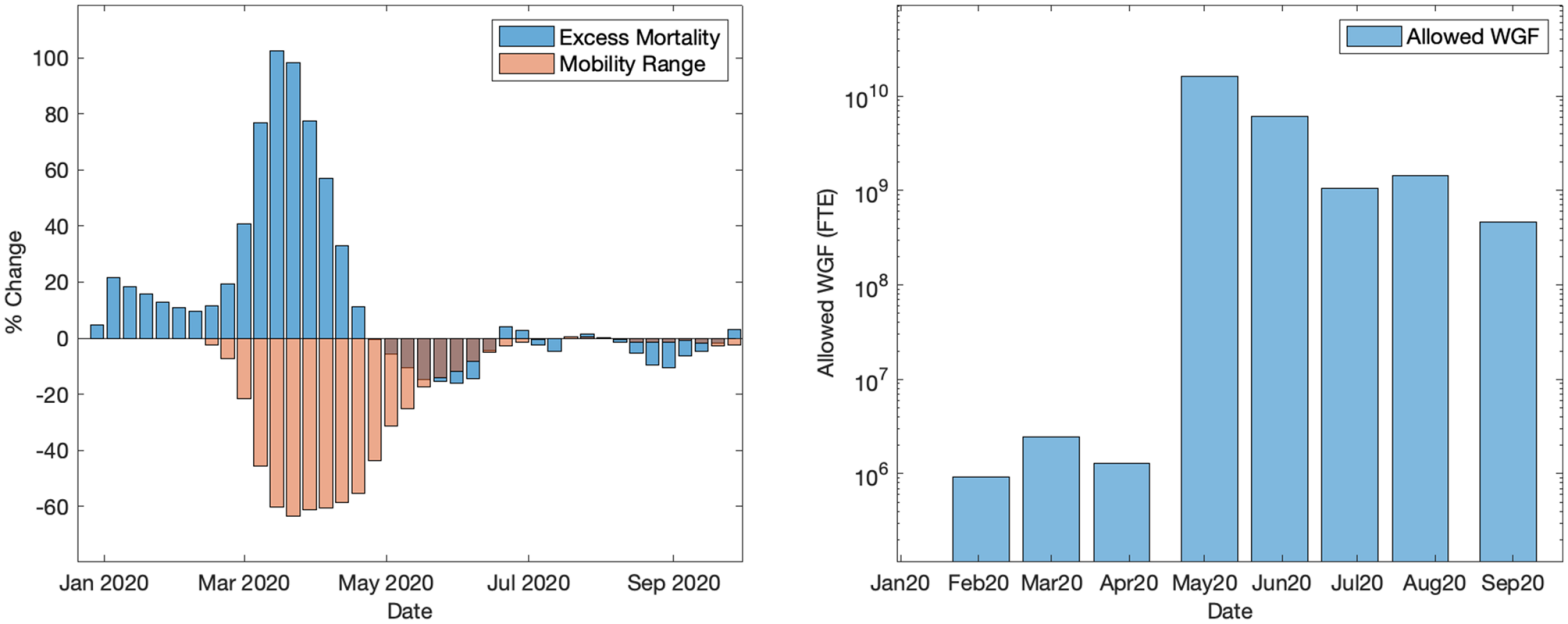
Wage Guarantee Fund, excess deaths and mobility range over time. The subplot on the left displays the average number of excess deaths per week and weekly average mobility changes. The subplot on the right shows the trend over time of the monthly average amount of allowed working hours covered by the Wage Guarantee Fund. The two-week moving average is reported for excess deaths and mobility range.

Figure 1 shows the temporal evolution of the main variables of interest in our empirical analysis. On the right-hand side, Fig. 1 shows the monthly average Wage Guarantee Fund (in terms of allowed working hours), while the panel on the left depicts the weekly average number of excess deaths and the weekly average drop in mobility over a time window spanning from January to October 2020. By the end of February, mobility drops significantly (up to 60%) while the number of excess deaths almost simultaneously shows a sharp increase. From the end of March on, we observe a rapid decrease in excess deaths following strict confinement measures. The same does not happen to the Wage Guarantee Fund: in the post-lockdown period, the average allowed Wage Guarantee Fund keeps values three orders of magnitude greater than in previous months. National public policies have had a remarkable impact on labor market flows. According to recent estimates, if measures like the extension of wage supplementation schemes together with firings freeze and financial support for firms had never been issued, there would have been 600 thousand more firings in 2020 because of the pandemic crisis^13^.

### Impact of restrictions on mortality and unemployment risk

Mobility restrictions have been enforced in many counties to curtail the diffusion of COVID-19 and reduce excess deaths. However, the diffusion of COVID-19 cases has also induced people to reduce non-essential movements spontaneously. To overcome potential endogeneity issues concerning our main variables of interest (excess deaths and furlough workers), we exploited the exogenous variation in rainfall patterns across geographies and over time as an instrument for the contraction of human mobility in Italy during the COVID-19 crisis.

Table 1 displays the main results from the instrumental variable model (2). Columns (1) and (2) show coefficient estimates obtained by regressing the logarithm of excess deaths on a one-month lagged change in mobility. Results have been estimated on the longitudinal dataset comprising monthly observations from March 2020 to October 2020 on 107 Italian provinces (NUTS 3 regions). Model specification in column (1) controls for time-related effects with the *Lockdown* dummy variable, which takes value 1 in those months when the first national lockdown was in force in Italy: March, April and May.

**Table 1:** Instrumental variable regression results for excess deaths and WGF.

	$\text{ ln } \text{ Excess } \text{ Deaths}_{it}$				$\text{ ln } \text{ Wage } \text{ Guarantee } \text{ Fund } \text{ FTE}_{it}$			
	Main regression		Robustness check		Main regression		Robustness check	
	(1)	(2)	(3)	(4)	(5)	(6)	(7)	(8)
	IV	IV	IV	IV	IV	IV	IV	IV
$\text{ Mobility } \text{ range}_{i(t-1)}$	0.667***	0.954***	0.491***	0.047*	− 10.133***	− 12.452***	− 5.218***	− 3.109***
	(0.160)	(0.300)	(0.061)	(0.028)	(0.864)	(2.142)	(0.242)	(0.174)
Lockdown	0.359***		0.300***		− 2.829***		− 1.564***	
	(0.058)		(0.032)		(0.257)		(0.126)	
Observations	846	846	855	855	611	611	618	618
Root MSE	0.212	0.327	0.204	0.225	1.350	2.546	0.981	1.243
Instrumental variable(s)	Rainfall	Rainfall	Share essentials Betweenness	Share essentials Betweenness	Rainfall	Rainfall	Share essentials Betweenness	Share essentials Betweenness

Columns from (1) to (4) and from (5) to (8) display IV regression estimates for the logarithm of excess deaths and the Wage Guarantee Fund, respectively.

Robust standard errors are reported in parentheses. All models control for NUTS 3 region-specific fixed effects. Specifications in columns (1), (3), (5) and (7) control for time trends with the Lockdown dummy, which takes value 1 when the first countrywide lockdown was in force (March, April, May).

Estimates in columns (1)–(2) and (5)–(6) are obtained instrumenting for mobility range by the share of consecutive rainy days recorded in a month. As a robustness check, model specifications in columns (3)–(4) and (7)–(8) employ both the time-varying share of essential residents and NUTS 3 regions’ betweenness centrality as excluded instruments for mobility range. All IVs have been lagged by one month to be in line with the endogenous regressor. First stage and reduced form results are reported in SI (see S3).

For comparative purposes, section S3 in SI Appendix displays fixed-effects estimates obtained without employing any instrumental variable, still controlling for NUTS 3 region-specific and time-specific fixed effects.

*p < 0.10 , **p < 0.05, ***p < 0.01.

We find a positive and statistically significant relationship between a change in mobility and excess deaths, which holds true when we control for time trends related to the enforcement of the countrywide lockdown. Column (1) shows that excess deaths decrease by around 0.6 percent at time *t* if mobility drops by one percent in the previous month. In the first stage regression, rainfall has an expected negative and statistically significant effect on mobility (see SI Appendix, Sect. 3—Regression tables).

Concerning the consequences of reducing mobility flows on employment risk, in the analysis of the Wage Guarantee Fund we use the same instrumental variable model in (2), exploiting the monthly share of consecutive rainy days as an exogenous instrument. First stage results confirm the negative effect of rainfall on mobility (see SI Appendix, Sect. 3—Regression tables). Model specifications in columns (5) and (6) have been estimated on the period going from March to August 2020, according to the wage guarantee fund data availability.

Results in column (5) show a negative and significant effect of mobility on the dependent variable: a 1% percent drop in mobility at time $(t-1)$ corresponds to a $10.1\%$ increase in allowed Wage Guarantee Fund in the following month. This implies that the enforcement of national policies was effective in curtailing the contagion, discouraged mobility flows and fostered the use of wage compensation schemes provided by law (Decree Law No. 18/2020 of 17 March 2020) to support workers.

As a robustness check, all model specifications have been estimated instrumenting for mobility with the NUTS 3 region’s betweenness centrality in the mobility network, and with the share of labor force employed in “essential” industries (columns (3)–(4) and (7)–(8)). Regression outputs confirm our findings (see methods and Sect. 3 of SI).

**Figure 2:**
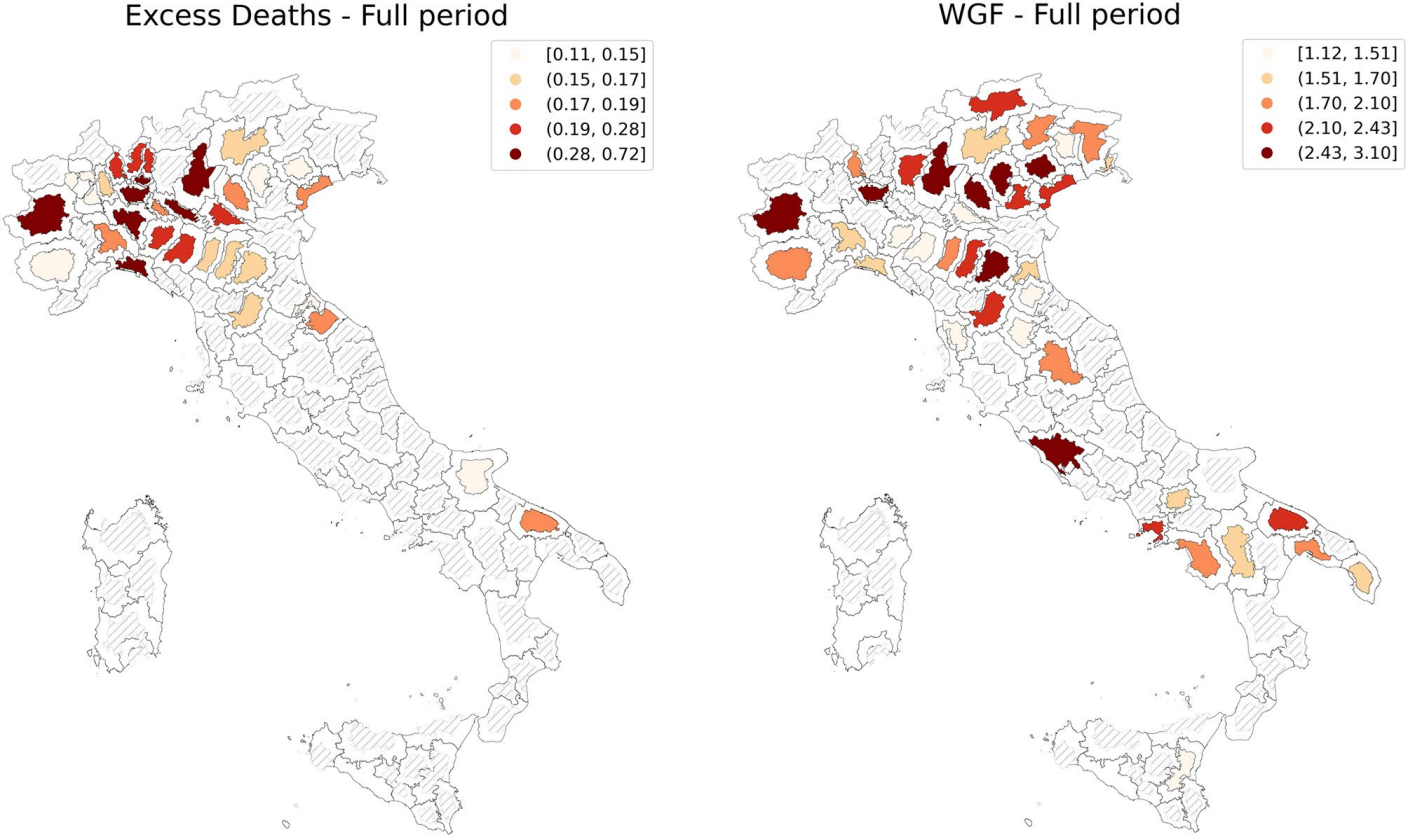
Graphical representation of the NUTS 3 region-specific fixed effects estimated through model (2). The color intensity of NUTS3 regions (Italian provinces) is proportional to the associated fixed-effect coefficient: darker colors denote higher coefficients. Each region in the plot is rescaled according to the average change in mobility that occurred between March and April 2020. The map on the left displays NUTS 3 specific fixed-effects estimates obtained when regressing the logarithm of excess deaths on mobility range (Table 1, column 1); the right-hand picture shows fixed-effects estimates when the Wage Guarantee Fund is the dependent variable (Table 1, column 5).

A comprehensive view of the impact of restrictions is observed in Fig. 2, which provides a graphical representation of NUTS 3 region-specific fixed effects estimates obtained from the main equation of the instrumental variable model (Table 1, columns (1) and (5)). The intensity of the color filling each territorial unit on the map is proportional to the associated fixed-effect coefficient: darker colors express higher coefficients. NUTS 3 regions whose coefficients are not statistically significant are in grey. The borders of the regions have been re-scaled according to the average mobility contraction experienced during the lockdown period.

Considering the excess mortality, the left panel of Fig. 2, highlights the NUTS 3 regions explaining a higher increase in the dependent variable in the whole period (once controlling by mobility and lockdown-related time trends). The most affected regions are located in the northern part of Italy. This also reflects the uneven spread of the disease across Italian regions^14^ during the first epidemic wave.

The map appears to be less polarized when considering the Wage Guarantee Fund (Fig. 2, right-hand side). Even if the observable pattern is different, we still observe that higher coefficients are concentrated in the north of the country and in the main urban areas located in central and southern Italy.

## Discussion

### Implications for the vaccination campaign

Our empirical analysis suggests prioritizing the areas where mobility restriction had a higher impact on the unemployment risk. Mobility has a causal effect on mortality and the number of infected people. To mitigate the health impact of the epidemics is essential to reduce the health risk of those essential workers that contribute to increasing mobility at the regional level. Therefore, essential workers and workers not eligible for remote working should be prioritized since they increase mobility, thus inducing higher excess mortality.

When considering the other categories, we find that, under the public health point of view, they are composed of substantially equivalent persons. The priority, in this case, should be driven by socio-economic considerations. To this aim and we suggest putting in a higher priority those workers having a higher unemployment probability or a higher risk of losing their job, increasing the likelihood to return to work. We propose to put in higher priority the areas where the observed increase in allowed WGF is stronger.

Referring to Fig. 2, on the right panel, we observe that the increase in the number of WGF allowed hours is not uniform, showing a consistent variability among Italian NUTS 3 regions. Colour intensity reflects the intensity of the fixed effects coefficients as from the IV model (Table 1, column (5)). Leveraging on this effect, we propose to rank the regions according to the intensity of their fixed effects, where higher values lead to those most in need of wages supplementation schemes.

**Figure 3:**
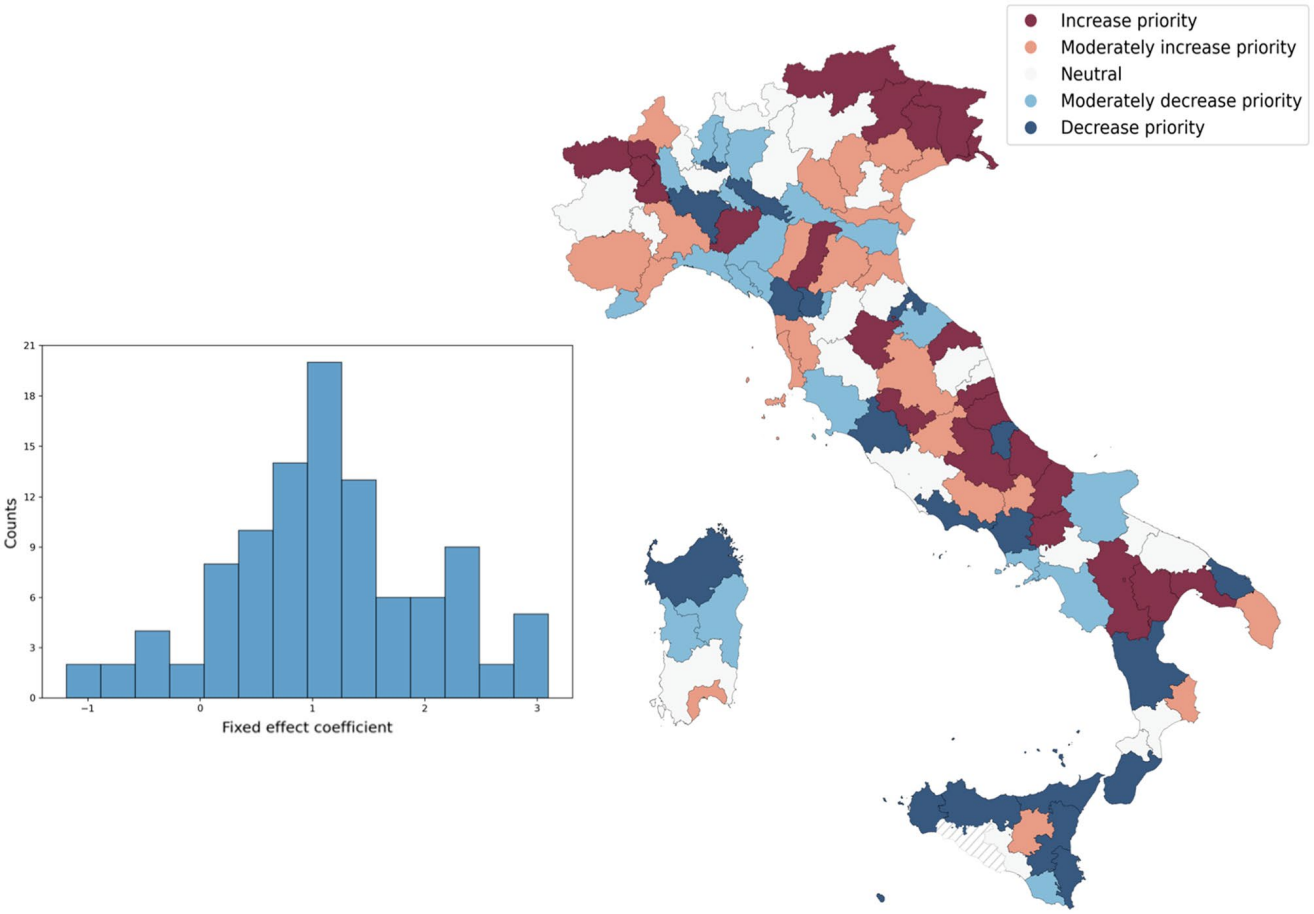
Left panel: Histogram of the Fixed effect values for WGF in the different Italian provinces (NUTS-3 regions); Right panel: Adjusted prioritization policy according to the ranking difference between WGF fixed effects and number of workers.

Such criterion should be more effective from the socio-economic point of view, and to show it, we compare the proposed allocation with a benchmark based on working population (WP). Each province *i* is ranked according to the two criteria explained above, and we indicate with $R^{WP}_i$ and $R^{WGF}_i$ the position of the region *i* in the Working Population and Wage Guarantee Fund criteria. To highlight possible inequalities, we compare the criteria by subtracting WP to WGF rankings:

1 $$\begin{aligned} \Delta _i^{WGF}= R^{WGF}_i - R^{WP}_i \end{aligned}$$

where $\Delta _i^{WGF}$ is the difference in the ranking positions between *WGF* and the working force ranking. The distribution of $\Delta$ is reported in Fig. 3. The intensity of the color is proportional to $\Delta _i^{WGF}$. Areas in light colors between blue and red tones are similar according to both criteria ($\Delta \sim \pm 10$); they are therefore equivalent under both criteria. Provinces in blue and dark blue shades correspond to NUTS 3 regions having $\Delta _i^{WGF}<0$; then they would be disadvantaged in the case of WP criterion. On the other hand, provinces in red and dark red shades ($\Delta >0)$, would be disadvantaged by distribution criteria based on public expenditure (WGF).

## Conclusions

We analyze the impact of human mobility on excess mortality and the use of furlough schemes in Italy. The impact of lockdown is found in low-income areas (as in^15^), but it also appears significant in the productive regions of northern/center Italy and in and urban areas of southern Italy. This is explained by the fact that the lockdown has had a greater impact on people in the low-income bracket in urban areas, who became vulnerable and at greater risk of marginalization. To mitigate the unemployment risk, we assume that safe return-to-work will be possible for vaccinated workers, reactivating mobility and restoring full production capacity. This is because the negative health consequences of human mobility will be neutralized. To this aim, we propose a vaccine prioritization policy of the health and active share of the population in two stages. First, access to vaccination should be guaranteed to essential workers and those not eligible for remote working. Then, return-to-work should be facilitated for the beneficiaries of wage guarantee schemes and workers facing higher unemployment risks. This will be beneficial both in terms of a reallocation and more efficient use of public funds and in terms of reduction of potential job losses. It is important to highlight that our recommendations refer to the phase of the vaccination campaign when vulnerable categories, according to the national strategic plan, have already been vaccinated and immunized against the virus.

The proposed strategy puts in advantage those workers employed in the areas in which wage integration measures have been used more, allowing them to come back sooner to a safe workplace (not taking into account potential market labor flows -especially firings- which could occur when public policies issued to increase employment protection will cease), triggering a gradual economic recovery. The expected benefit of this policy can be interpreted mostly in terms of a gradual resumption of most economic activities and in terms of potential alternative allocations of public funds. We recall that, according to the European Commission^16^, the Italian government has committed around 19 billion euros to cover wage supplementation schemes. Under the public health point of view, results show the existence of a positive and significant association between ’one month-lagged’ mobility changes and the excess deaths recorded: a one percent drop in mobility (w.r.t. the baseline) explains a 0.6% drop in the number of excess deaths in the following month.

Our results are relevant for an international audience as well, since similar employment protection measures have been issued by European governments as a response to the pandemic. Short-time furlough schemes meant to support the firms affected by the crisis have been introduced or extended in Europe^16^. European Union member states are allowed to ask for European funds in order to cover such employment protection measures: financial support in the form of loans granted on favorable terms is provided under the SURE instrument (temporary Support to mitigate Unemployment Risks in an Emergency).

With regards to the short availability of the vaccines and their optimal distribution, the results presented are particularly relevant in middle and low-income countries, where the share of people fully vaccinated against COVID-19 is significantly lower with respect to high-income countries, with percentages ranging between 1 and 30%^17^.

Given the heterogeneous impact of the pandemic on local employment^18^ and across sectors^19^ this criterion could be extended to address even those workers who lost their job because of the pandemic crisis: immunity against COVID-19 could facilitate applying for and starting a new job more safely.

Future research will be devoted to the understanding of how the mobility patterns may influence the employment risk and the vaccination campaigns in other countries and across different sectors of the economy.

## Methods

### Data availability statement

Regarding Facebook human mobility, all data are provided under an academic license agreement with Facebook through its “Data for Good” program (https://dataforgood.fb.com/tools/disease-prevention-maps/). Facebook releases data upon request to nonprofit organizations and academics. Concerning the socio-economic variables and the mobility matrix that we have used to validate the pre-lockdown Facebook mobility data, they are all publicly available through the Italian Statistical Office ISTAT (http://dati.istat.it/), except for the WGF allowed hours that are available through INPS, the National Social Insurance Agency (https://https://www.inps.it/OpenData/).

### Human mobility data

We analyzed mobility between NUTS-3 regions (corresponding to the Italian provinces) based on “Disease Prevention Maps” provided by Facebook through its “Data for Good” program^20^. These maps consist of anonymous and aggregated information of Facebook users collected starting from their mobile phones with GPS enabled. Data do not directly indicate numbers of individuals traveling but are constructed by Facebook with proprietary methods (including mechanisms to ensure privacy protection) to provide an index that correlates with the actual movement of people. We collected data relative to movements between Italian NUTS-3 regions (namely “movement between administrative regions”) from February 23 to October 1st 2021. Mobility contraction has been estimated by using the Facebook “Movement Range” data, providing, for each NUTS-3 region, an estimate of the average daily contraction of mobility with respect to the pre-lockdown period. Data are not publicly available; Facebook provided them under an academic license agreement. The reader is referred to Sect. 4 of the supplementary information for a comparison and robustness check between Disease Prevention Maps data and the mobility census data provided by ISTAT.

### Excess mortality data

Following^21^, we considered the excess mortality data as representative of the epidemic spreading. Such an assumption is needed to overcome the potential issues related to the endogeneity of testing policies (showing different criteria, especially during the first wave of the epidemics), hospital capacity, and difference in death classification at the local level. Excess mortality data are available through the website of the National Statistical Office (ISTAT). For each municipality, data report the daily number of deaths for the years 2015–2020. Excess mortality has been computed by aggregating data at NUTS-3 level (Italian provinces) and comparing the number of deaths with the 2015–2019 average: it expresses the difference between the number of deaths recorded in 2020 and the average number of deaths that occurred in the previous five years. The temporal aggregation has been set at the weekly level.

### Furlough schemes

Furlough schemes are measured as the number of Wage Guarantee Funds allowed hours to proxy the impact of national policies and imposed shutdowns on private sector economic activities. Data on WGF allowed hours are available at the National Social Insurance Institute (INPS). Monthly allowed hours are aggregated at NUTS-3 region level (Italian provinces) and cover both the economic sectors (ATECO) and the type of worker furloughed (worker or employee).

### Rainfall

Daily weather data covering the year 2020 have been collected from the weather forecast website ilmeteo.it. Relying on these data, we computed the share of consecutive rainy days recorded in a month over 2020 at Italian provinces (NUTS 3 level).

### Betweenness centrality

Betweenness centrality^22^ has been estimated on the directed and weighted network computed from the Facebook data accounting for the mobility between NUTS-3 regions (namely: mobility between administrative regions). A sample picture of the mobility network is provided in Sect. 6 of the supplementary material (Fig. S5).

### The instrumental variable model

Empirical estimates concerning the effect of mobility patterns on the Covid-19 death toll and wage integration schemes are likely to be affected by endogeneity issues, potentially in the form of reverse causality or omitted variable bias^23–25^. To disentangle endogeneity and provide consistent estimates, we leveraged the exogenous variation in weather patterns across geographies and over time as a source of exogenous shocks to human mobility. The employment of weather data in econometric models is widely observed in the empirical literature^7–9,26–28^: given their exogenous nature, weather metrics have been exploited in IV frameworks to infer causal relationships between explanatory and outcome variables.

To perform our analysis, we computed the share of consecutive rainy days (at least two in a row) recorded in a month to be used as an instrument. The choice to exclude “isolated” rainy days from the count is meant to leave out transient phenomena, which potentially have no or just a little impact on human mobility. This actually improves the relevance of the instrument.

In order *Rainfall* to be a valid instrument, specific conditions must be satisfied: the metric has to be exogenous (i.e., uncorrelated with the error term) and must be correlated with the endogenous regressor, namely mobility range. Concerning the first requirement (the exogeneity condition), we argue that *Rainfall*, a naturally occurring phenomenon, is not related to the unobservable factors affecting the dependent variables (wage guarantee fund hours and excess deaths). Relying on the exclusion restriction assumption, we state that an induced change in mobility must be the main channel through which weather conditions (*Rainfall* specifically) may affect the use of furlough schemes and the number of excess deaths. We reasonably accept the latter hypothesis by assuming weather conditions to have a very negligible impact on the economic trends of a developed country like Italy (which in turn could lead to a higher request for furlough schemes) as well as a negligible effect on the number of excess deaths which are primarily due to the pandemic. Additionally, we formally ran a test to rule out concerns due to *Rainfall* potential weakness when estimating the first-stage regression. First stage statistics confirm the relevance of our instrumental variable (see sub-section “Endogeneity and IVs relevance test” below and SI Appendix, Sect. 3—Regression tables).

The first stage and main equations for the IV model are given by

2 $$\begin{aligned} Mob.Range_{i(t-1)}= & {} \pi IV_{i(t-1)} + \gamma Lockdown_{t} + pv_i + \eta _{it} \nonumber \\ ln(y)_{it}= & {} \beta Mob.Range_{i(t-1)} +\delta Lockdown_{t} + pv_i + \varepsilon _{it} \end{aligned}$$

where $pv_i$ denotes NUTS 3 region-specific fixed effects, which allow to control for time-invariant unobserved characteristics of Italian provinces. Both stages include the *Lockdown* dummy which takes value 1 in the months of March, April and May to control for potential time-related effects due to the enforcement of the first countrywide lockdown. In the main equation, the logarithm of the dependent variable is regressed on a month lagged value of the explanatory variable *Mobility Range*: usually, a delay of about one month occurs between the time in which a firm requires the wage supplementation schemes and the time it is officially authorized and recorded^11^. Similarly, changes in the mobility induced by restriction measures are followed with some delay by a decrease of COVID-19 deaths^29^. To be in line with the instrumented variable the IV has been lagged by one month.

We perform a GMM estimation of the coefficients of the model. Concerning the interpretation of parameter $\beta$, a unitary increase in mobility implies a $(\beta *100$ )% variation in the dependent variable (since ’Mobility range’ is not expressed in percentage points a unitary change in mobility means actually a 100$\%$ change).

### Robustness checks

To corroborate our results, we performed a robustness check instrumenting for mobility by the time-varying share of essential residents (the share of the labor force employed in economic sectors designated as essential according to the provisions of Prime Ministerial Decrees) and by the time-varying betweenness centrality of NUTS3 regions. Following^23,24^, we rely on the assumption that the centrality of a province in the mobility network and the share of people employed in essential industries have an impact on excess deaths just through mobility flows. A similar argument applies to wage guarantee schemes.

Concerning the impact of a contraction in mobility on furlough schemes, we still observe a negative and statistically significant relationship between mobility range and wage guarantee fund allowed hours, consolidating our previous results (Table 1, column (7)–(8)). Once again, regression outputs are robust to the inclusion of the lockdown dummy. Estimates from the first stage show that more central NUTS 3 regions and regions with a lower fraction of essential working residents have experienced a higher drop in mobility (see SI, Sect. 3—Regression tables). About the effectiveness of mobility restrictions in curtailing the contagion, coefficients in columns (3) and (4), Table 1, confirm that a contraction in mobility implies a drop in the number of excess deaths in the following month.

Finally, as a further check, model specifications (1)–(2)–(5)–(6) have been estimated employing an alternative measure for rainfall share, which computes the actual number of rainy days in a month, including isolated ones. We observe just a marginal change in coefficients estimates but lower first-stage F-statistics; thus, the choice of including just consecutive rainy days in the metric actually improved IV relevance. The interested reader can find the results of a fixed effect panel model without IVs and all the results for alternative model specifications in the SI Appendix.

### Endogeneity and IVs relevance tests

We tested the endogeneity of the main explanatory variable mobility range, running a C test of endogeneity: the value of the test statistic lets us reject the null hypothesis that the regressor is exogenous. This is true for every model specification in Table 1 (all *p*-values fall below 0.013).

About the relevance of the instruments, first stage F-statistics suggest that IVs are strong: when instrumenting by rainfall, F-statistics range from 23.69, model specification (6), to 82.04, model specification (1). When we instrument by NUTS 3 regions betweenness and share of essential residents F-statistics range from 292.32, model specification (3), to 498.91, model specification (4).

## Supplementary Information

Supplementary Information.
