# Supplementary material for: COVID-19 vaccination and unemployment risk: lessons from the Italian crisis
Source: Sci Rep. 2021 Sep 17;11:18538. doi: 10.1038/s41598-021-97462-6 (PMC8448894; doi:10.1038/s41598-021-97462-6)
Supplement: Supplementary file 1 — Supplementary Information. [file 41598_2021_97462_MOESM1_ESM.pdf]

Supplementary Information for

COVID-19 Vaccination and unemployment risk: Lessons from the Italian crisis

Valentina Pieroni, Angelo Facchini, Massimo Riccaboni

Corresponding Author name.
E-mail: angelo.facchini@imtlucca.it

This PDF file includes:

Supplementary text
Figs. S1 to S5 (not allowed for Brief Reports)
Tables S1 to S6 (not allowed for Brief Reports)
SI References

Supporting Information Text

1. The instrumental variable approach

To provide insights about the impact of mobility contraction on furlough schemes and excess deaths we implemented an instrumental variable model accounting for Italian NUTS 3 regions' unobserved heterogeneity.

As already pointed out in previous scientific works, mobility is likely to be endogenous with variables strictly related to the spread of the disease, such as the number of COVID-19 cases or excess deaths (1–3). A potential reversed causality issue may affect the estimates since mobility flows are adjusted when people observe an increase (or decrease) in the spread of COVID-19.

A similar argument applies to the relationship between mobility and the amount of the Wage Guarantee Fund. Reduced mobility can explain an increase in the Wage Guarantee Fund since the enforcement of containment measures meant to discourage mobility and limit social interactions could foster the use of wage guarantee schemes to reduce physical proximity in the workplace. However, temporary suspension of working activities could itself explain a further drop in commuting flows. This could be the most intuitive way to interpret the relationship between mobility and furlough schemes but is not the only one, as mobility could impact the Wage Guarantee Fund even through different channels. If fewer people move because of containment rules or fear of contagion, we may observe a decline in the demand for goods and services by final consumers. In turn, employers may opt for a temporary reduction of working time and ask for wage compensation schemes to cope with a contraction in the demand even as a potential effect of consumer substitution patterns (4).

Our empirical approach leverages the exogenous variation in weather patters over time and across geographies to overcome endogeneity concerns. We instrumented for mobility by the share of consecutive rainy days recorded in a month over 2020 at Italian NUTS 3 level, namely *Rainfall share*. We built the metric excluding from the count "isolated" rainy days not belonging to a series comprising at least 2 consecutive rainy days.

Rainfall shocks have been widely used as instruments in econometric settings affected by endogeneity, as in several scientific works investigating the relationship between economic shocks and conflicts (5–8), where income growth is instrumented by the exogenous variation in rainfall. A connection between weather shocks and income can be observed and theoretically justified for those countries whose economy depends largely on rain-fed agriculture. However, the availability of developed irrigation infrastructures together with new agriculture technologies and a more relevant contribution of the industrial sector to the national (and regional) economy, make income growth less sensitive to weather shocks. In the specific case of Italy, we assume weather conditions to have a very negligible impact on economic activities, therefore we claim that the effect of rainfall on furlough schemes does not go through a potential shock on economic activities but through an induced variability in human mobility. Similarly, we assume human mobility patterns to be the only channel through which weather shocks can potentially have an impact on the number of excess deaths recorded during the pandemic.

To corroborate our findings, we performed a robustness check exploiting two alternative instruments for mobility.

The choice of the first instrument is inspired by (1). Looking at the provisions of Prime Ministerial Decrees issued between March and May[*] we computed the time-varying share of essential residents (*Share Essentials*) in each NUTS 3 region, that is, the share of labor force which was allowed to move during the first national lockdown since employed in economic sectors designated as essential by the Italian government. The share of authorized employees has been multiplied by the 2019 employment rate of Italian provinces [†] to proxy the share of essential workers of NUTS 3 regions.

As a second instrumental variable (IV), we computed the time-varying betweenness centrality (9) of Italian NUTS 3 regions in the mobility network built on Facebook data movement between administrative regions. A low value of the betweenness centrality is a proxy of Italian NUTS 3 regions' remoteness (2). Mobility range measures the average reduction of mobility within an administrative region, in contrast, the betweenness centrality looks at the whole network, in a global perspective, providing a ranking of Italian NUTS 3 areas based on their importance of bridging different regional mobility systems. Based on this, we assume this quantity to be less or even not susceptible to changes in the number of fatalities or the number of Wage Guarantee Fund allowed hours at a local scale (i.e. changes referring to the single NUTS 3 region).

Following the argument in (1) and (2), we assume that the centrality of a territorial unit in the mobility network and the share of people employed in essential industries have an impact on excess deaths just through mobility flows. A similar argument applies for the Wage Guarantee Funds allowed hours.

We estimated three specifications of the model, as displayed in the following section (table S3 for excess deaths and S5 for the Wage Guarantee Fund): the first one employs only the betweenness centrality as an IV, the second one includes just *Share Essentials*, the third model uses both variables as excluded instruments.

[*] Our references are Dpcm March 11, Dpcm March 22, Dpcm April 1, Dpcm April 10, Dpcm April 26 and Dpcm May 17, 2020.

[†] Source of employment data is ISTAT (Italian National Institute of Statistics) Labour Force Survey.

2. Variables used

Table S1. Data description

	Mean	Std. Dev.	Min.	Max.	Obs.
W.G.F. FTE	14676.54	30937.9	0	355018.5	856
Excess deaths	48.884	264.510	-478.2	5181.4	1070
Mobility Range	-0.185	0.199	-0.688	0.155	1177
Rainfall share	0.197	0.158	0	0.774	1274
Betweenness	0.030	0.065	0	0.581	855
Share Essentials	60.459	13.131	23.746	79.2	1284

Descriptive statistics for the main variables included in the econometric model. The Wage Guaranetee Fund (W.G.F.) is expressed in full time equivalent (FTE) units.

3. Regression tables

In this section we provide further details on regression outputs and statistics.

Table S2 reports IV estimates obtained regressing the Wage Guarantee Fund (in FTE units) and excess deaths on mobility range. Model specifications in columns (1) and (2) control for time trends including the Lockdown dummy variable. As a further check, estimates have been repeated controlling for time-related effects with a set of dummy variables defined for each month. The alternative specification yields a positive and statistically significant coefficient for mobility when excess deaths is the dependent variable, while, concerning the Wage Guarantee Fund, mobility's coefficient loses statistical significance, potentially because of the short time horizon covered by wage guarantee fund data. Results from this check are available upon request.

More details about robustness checks outputs are displayed in tables from S3 to S6.

Table S2. Wage Guarantee Fund and Excess Deaths: first stage and reduced form regressions

	ln Wage Guarantee Fund FTE$_{it}$			ln Excess Deaths$_{it}$		
	(1)<br>Main	(1.f)<br>First Stage	(1.r)<br>Reduced Form	(2)<br>Main	(2.f)<br>First Stage	(2.r)<br>Reduced Form
Mobility range$_{i(t-1)}$	-10.133***			0.667***		
	(0.864)			(0.160)		
Lockdown	-2.829***	-0.268***	-0.114	0.359***	-0.304***	0.155***
	(0.257)	(0.016)	(0.111)	(0.058)	(0.014)	(0.017)
Rainfall share$_{i(t-1)}$		-0.487***	4.931***		-0.436***	-0.291***
		(0.061)	(0.456)		(0.048)	(0.070)
Observations	611	611	611	846	846	846
Number Ids	104	104	104	107	107	107
Root MSE	1.350	0.200	1.405	0.212	0.177	0.208
First Stage F-Stat.	62.87			82.04		
NUTS3 regions FE	Yes	Yes	Yes	Yes	Yes	Yes

Robust standard errors in parentheses.

Columns from (1) to (1.r) and from (2) to (2.r) display IV regression estimates for the Wage Guarantee Fund and excess deaths respectively, when controlling for time-related effects with the Lockdown dummy variable. Results are obtained instrumenting for mobility range by the share of consecutive rainy days recorded in a month over 2020 at NUTS 3 level. The models control for NUTS 3 region-specific fixed effects.

* $p < 0.10$, ** $p < 0.05$, *** $p < 0.01$

Table S3. Robustness check: excess deaths regression results

	ln Excess Deaths$_{it}$			
	(1) FE	(2) IV	(3) IV	(4) IV
Mobility range$_{i(t-1)}$	0.321***	0.733***	0.492***	0.491***
	(0.042)	(0.201)	(0.060)	(0.061)
Lockdown	0.252***	0.374***	0.303***	0.300***
	(0.038)	(0.067)	(0.032)	(0.032)
Constant	6.231***			
	(0.007)			
Observations	856	855	856	855
Number Ids	107	107	107	107
Individual FE	Yes	Yes	Yes	Yes
Overall R^2	0.137			
Root MSE	0.188	0.215	0.204	0.204
First Stage F-Stat.		53.64	565.13	292.32
Hansen J stat.				1.550
Instrumental variable(s)		Betweenness	Share essentials	Share essentials<br>Betweenness

Robust standard errors in parentheses

Regression estimates are obtained on the panel comprising monthly observations from March 2020 to October 2020 on a cross-section of 107 Italian NUTS 3 regions. Model specification in column (2), employs the betweenness centrality as excluded instrument for mobiliy; specification in column (3) employs share essentials and column (4) shows results obtained using both time-varying IVs. For comparative purposes, column (1) displays fixed-effects estimates obtained assuming mobility to be exogenous.

* $p < 0.10$, ** $p < 0.05$, *** $p < 0.01$

Table S4. Robustness check: excess deaths first stage and reduced form regressions

[A] First Stage

	(2) Mobility range$_{i(t-1)}$	(3) Mobility range$_{i(t-1)}$	(4) Mobility range$_{i(t-1)}$
Betweenness$_{i(t-1)}$	-1.376***		-0.409***
	(0.188)		(0.150)
Lockdown	-0.286***	-0.102***	-0.102***
	(0.015)	(0.012)	(0.012)
Share essentials$_{i(t-1)}$		0.017***	0.017***
		(0.001)	(0.001)
Observations	855	856	855
Number Ids	107	107	107
Root MSE	0.180	0.120	0.119
Individual FE	Yes	Yes	Yes

[B] Reduced form

	(2) ln Excess Deaths$_{it}$	(3) ln Excess Deaths$_{it}$	(4) ln Excess Deaths$_{it}$
Betweenness$_{i(t-1)}$	-1.008***		-0.547**
	(0.283)		(0.265)
Lockdown	0.164***	0.253***	0.252***
	(0.018)	(0.025)	(0.025)
Share essentials$_{i(t-1)}$		0.009***	0.008***
		(0.001)	(0.001)
Observations	855	856	855
Number Ids	107	107	107
Root MSE	0.208	0.198	0.197
Individual FE	Yes	Yes	Yes

Robust standard errors in parentheses

First stage and reduced form results refer to model specifications displayed in columns (2), (3) and (4)of table S3.

* $p < 0.10$, ** $p < 0.05$, *** $p < 0.01$

Table S5. Robustness check: Wage Guarantee Fund regression results

	ln Wage Guarantee Fund FTE$_{it}$			
	(1)	(2)	(3)	(4)
	FE	IV	IV	IV
Mobility range$_{i(t-1)}$	-5.680***	-3.254***	-5.304***	-5.218***
	(0.192)	(0.748)	(0.242)	(0.242)
Lockdown	-1.673***	-1.051***	-1.576***	-1.564***
	(0.093)	(0.234)	(0.125)	(0.126)
Constant	8.273***			
	(0.019)			
Observations	619	618	619	618
Number Ids	104	104	104	104
Individual FE	Yes	Yes	Yes	Yes
Overall R^2	0.438			
Root MSE	0.890	1.099	0.978	0.981
First Stage F-Stat.		49.29	599.79	305.92
Hansen J stat.				9.961
Instrumental variable(s)		Betweenness	Share essentials	Share essentials<br>Betweenness

Robust standard errors in parentheses

Regression estimates are obtained on the full period covered by WGF data, spanning from March 2020 to August 2020. Model specification in column (2), employs the betweenness centrality as excluded instrument for mobiliy; specification in column (3) employs share essentials and column (4) shows results obtained using both time-varying IVs. For comparative purposes, column (1) displays fixed-effects estimates obtained assuming mobility to be exogenous.

* $p < 0.10$, ** $p < 0.05$, *** $p < 0.01$

Table S6. Robustness check: Wage Guarantee Fund first stage and reduced form regressions

[A] First Stage

	(2) Mobility range$_{i(t-1)}$	(3) Mobility range$_{i(t-1)}$	(4) Mobility range$_{i(t-1)}$
Betweenness$_{i(t-1)}$	-1.426***		-0.329**
	(0.203)		(0.159)
Lockdown	-0.249***	-0.068***	-0.069***
	(0.017)	(0.013)	(0.013)
Share essentials$_{i(t-1)}$		0.017***	0.017***
		(0.001)	(0.001)
Observations	618	619	618
Number Ids	104	104	104
Root MSE	0.203	0.130	0.130
Individual FE	Yes	Yes	Yes

[B] Reduced form

	(2) ln WGF FTE$_{it}$	(3) ln WGF FTE$_{it}$	(4) ln WGF FTE$_{it}$
Betweenness$_{i(t-1)}$	4.641***		-1.336
	(1.395)		(1.412)
Lockdown	-0.239*	-1.217***	-1.223***
	(0.126)	(0.149)	(0.149)
Share essentials$_{i(t-1)}$		-0.092***	-0.093***
		(0.007)	(0.007)
Observations	618	619	618
Number Ids	104	104	104
Root MSE	1.528	1.269	1.269
Individual FE	Yes	Yes	Yes

Robust standard errors in parentheses.

First stage and reduced form results refer to model specifications displayed in columns (2), (3) and (4) of table S5.

* $p < 0.10$, ** $p < 0.05$, *** $p < 0.01$

4. Facebook and census data

In this section we test how Facebook data represent a reliable approximation of the population commuting for work and study, i.e. excluding those that do not habitually move within the provinces and between the provinces. Our idea is to test this reliability by looking at the data provided by the Italian statistical office in 2011, i.e the last census data collection in Italy.

Both data are presented in the form of a origin destination matrix, where the links express the number of commuters who travel between and within provinces. In the case of ISTAT data, people move for study or work, in all time slots, and data are averaged over a period of one year. On the other hand, Facebook data account for the people moving daily, sampled at 8 hours intervals. In order to compare data, we averaged over the whole available period the Facebook data obtaining an averaged origin destination matrix. Our hypothesis is that the temporal averaging leads to a origin destination matrix accounting for the habitual commuting of the study and workforce.

Figure S1 shows the plot of the weight of the links accounted in both OD matrices. From a linear fit we find that Facebook data and ISTAT data are on average 1 to 7.5 ratio, confirming the data provided by (10). As a further test, we considered only the Facebook traffic recorded during the daytime (8AM-4PM), comparing it to the corresponding period of ISTAT data. Results are depicted in Figure S2, where the agreement of the two datasets is conserved. According to these results we could consider, although sampling a smaller part of the population, Facebook movement data reliable under the point of view of the characterization of the mobility of habitual commuters.

Fig. S1. Scatter plot of the commuting flows of ISTAT and Facebook mobility data.

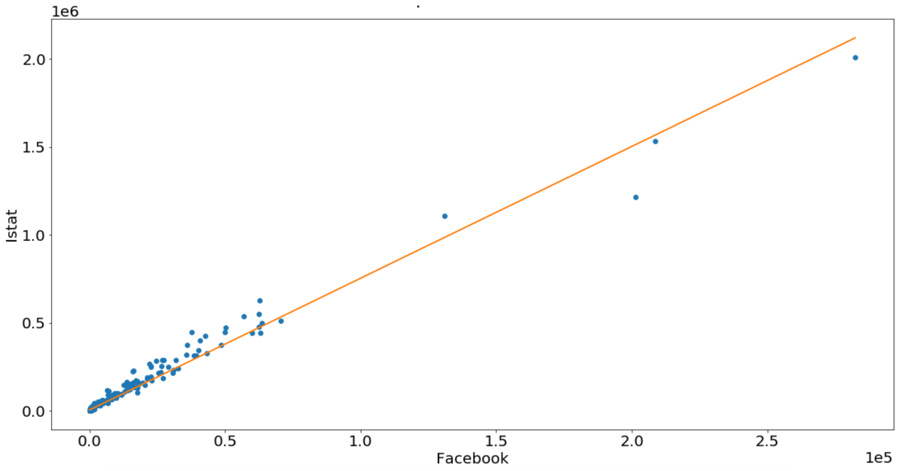

Fig. S2. Focus on daytime (8AM-4PM): Scatter plot of the commuting flows of ISTAT and Facebook mobility data. Fit 7.49, $R^2 = 0.97$

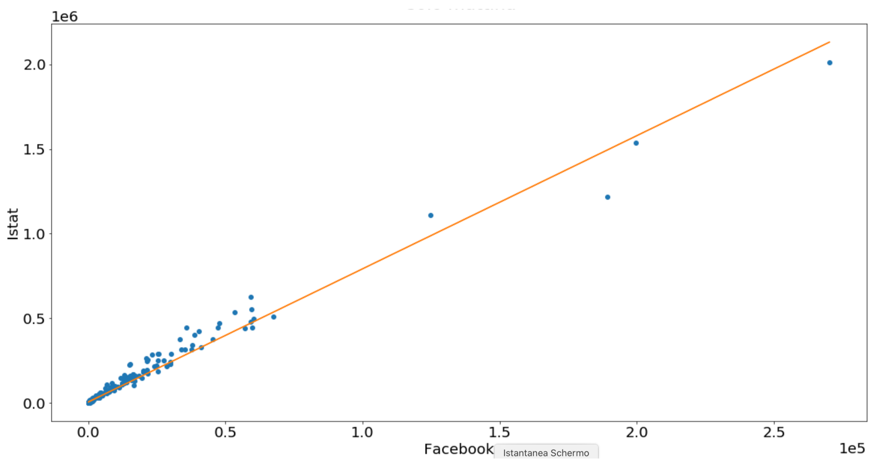

5. Maps and plots

Fig. S3. Monthly Wage Guarantee Fund allowed hours: January to April

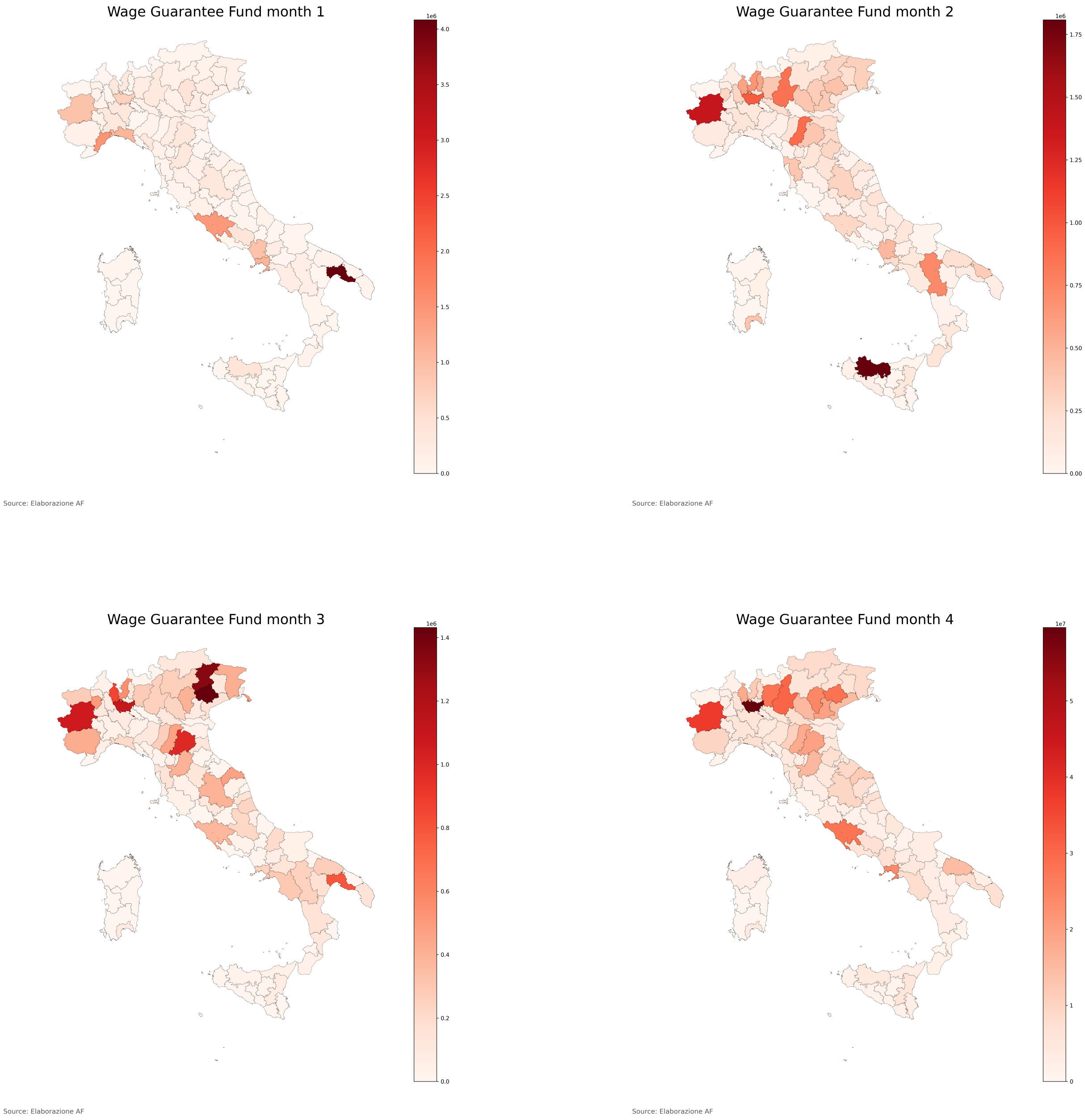

Fig. S4. Monthly Wage Guarantee Fund allowed hours: May to August

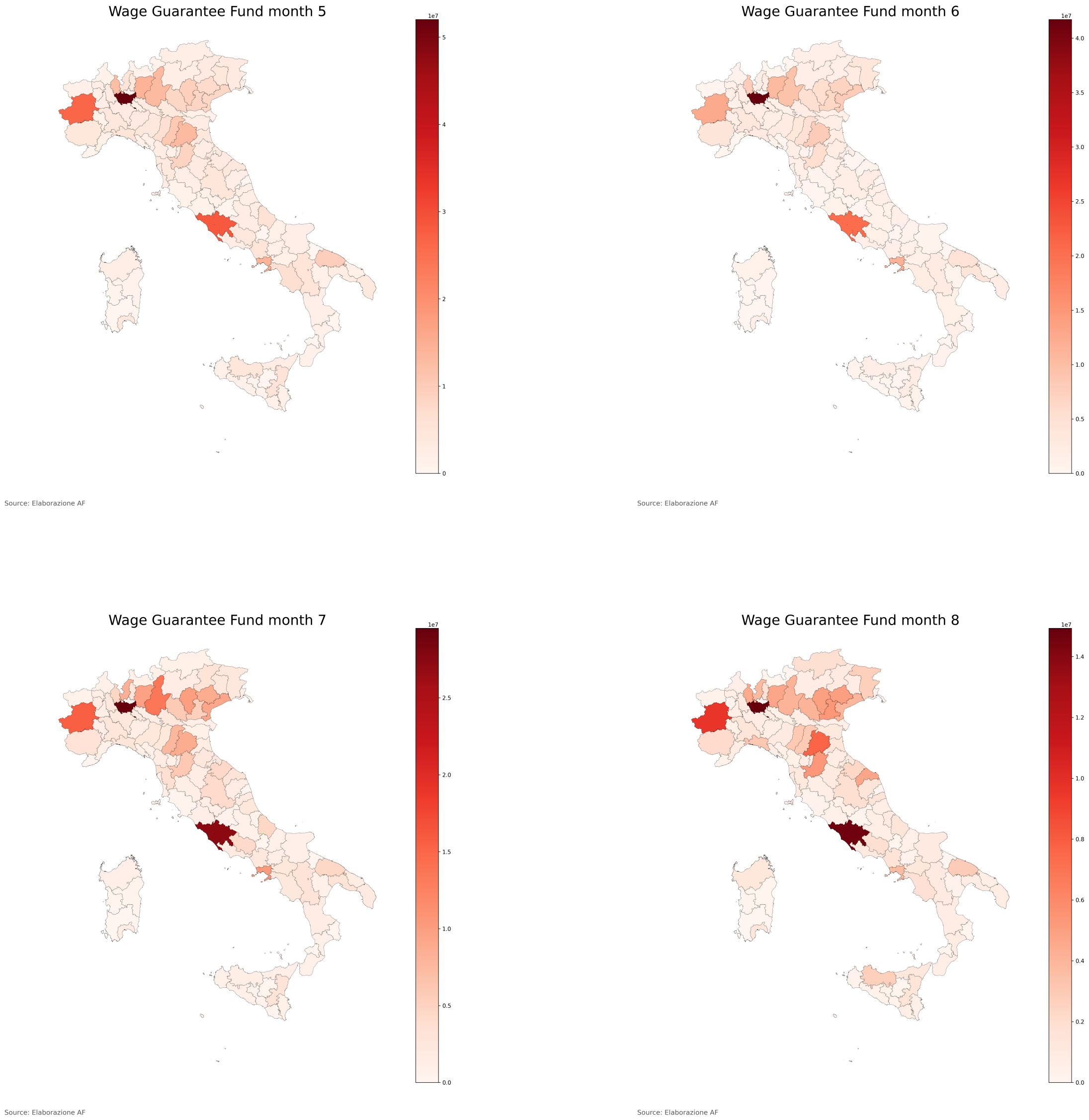

6. Network of the NUTS-3 level origin-destination matrix

The origin-destination matrix has been estimated starting from the data provided by the Facebook data for good repository. The resulting oriented and weighted network is depicted in figure S5. Node colour indicate the community of each node, node size is proportional to betweenness centrality and the intensity of the edge accounts for the intensity of the people flow between two regions.

Fig. S5. Directed and weighted network of the origin-destination matrix of NUTS-3 region. Average of the pre-pandemic period (Jan-Feb 2020).

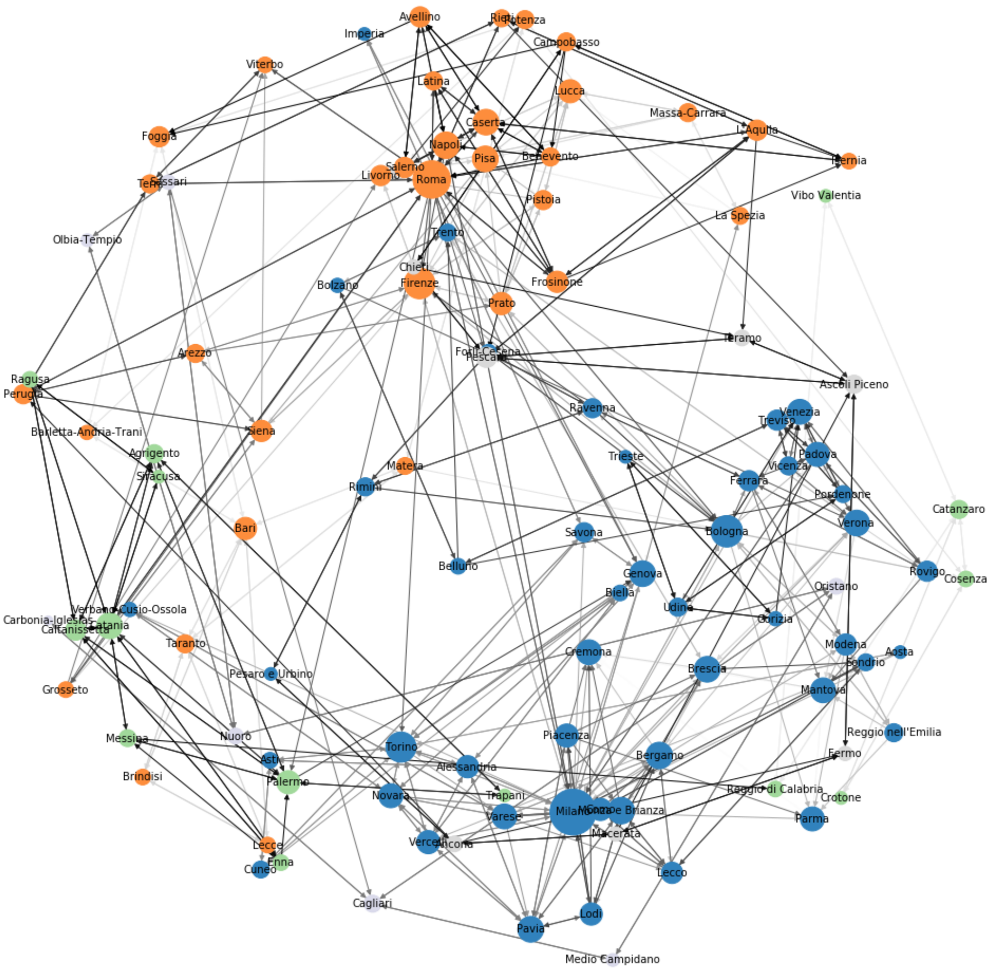

References

1. EL Glaeser, C Gorback, SJ Redding, JUE insight: How much does COVID-19 increase with mobility? Evidence from New York and four other U.S. cities. *J. Urban Econ.*, 103292 (2020).
2. A Krenz, H Strulik, The Benefits of Remoteness - Digital Mobility Data, Regional Road Infrastructure, and COVID-19 Infection. (2020).
3. N Borri, F Drago, C Santantonio, F Sobbrio, The 'Great Lockdown': Inactive Workers and Mortality by COVID-19. (2020).
4. A Goolsbee, C Syverson, Fear, lockdown, and diversion: Comparing drivers of pandemic economic decline 2020. *J. Public Econ.* **193**, 104311 (2021).
5. E Miguel, S Satyanath, E Sergenti, Economic shocks and civil conflict: An instrumental variables approach. *J. Polit. Econ.* **112**, 725–753 (2004).
6. H Sarsons, Rainfall and conflict: A cautionary tale. *J. Dev. Econ.* **115**, 62–72 (2015).
7. P Sandholt Jensen, K Skrede Gleditsch, Rain, growth, and civil war: The importance of location. *Def. Peace Econ.* **20**, 359–372 (2009).
8. AT Bohlken, EJ Sergenti, Economic growth and ethnic violence: An empirical investigation of hindu—muslim riots in india. *J. Peace Res.* **47**, 589–600 (2010).

9. M Newman, *Networks: An introduction.* (Oxford University Press), (2010).
10. G Bonaccorsi, et al., Economic and social consequences of human mobility restrictions under COVID-19. *Proc. Natl. Acad. Sci. United States Am.* **117**, 15530–15535 (2020).
